# Research on Broken Wire Based on a Fine Finite Element Model of Steel Strands

**DOI:** 10.3390/ma18133148

**Published:** 2025-07-02

**Authors:** Dongmei Tan, Yongfa Luo, Yu Tao, Yu Peng, Hao Luo, Baifeng Ji

**Affiliations:** 1 Sanya Science and Education Innovation Park, Wuhan University of Technology, Sanya 572000, China; jbfeng@whut.edu.cn; 2 School of Civil Engineering and Architecture, Wuhan University of Technology, Wuhan 430070, China; luoyongfa101@163.com (Y.L.); taoyu123456@whut.edu.cn (Y.T.); pengyu0706@whut.edu.cn (Y.P.); lh3390864306@whut.edu.cn (H.L.)

**Keywords:** seven-wire steel strand, finite-element beam-element model, symmetrical broken wire, asymmetric broken wire

## Abstract

In order to study the mechanical properties of seven-wire steel strands after wire breakage failure, this study is based on the established finite-element beam-element model of seven-wire steel strands and analyzes two failure forms of symmetric wire breakage and asymmetric wire breakage. The stress redistribution pattern, recovery length, and parameter influences (temperature: 0–40 °C; friction coefficient: 0.15–0.30; torsion angle: 4–7°) are analyzed. The results show that broken wire damage will cause the stress of the intact steel wire to increase rapidly, increasing the risk of fracture of the intact steel wire. The recovery length will shorten with the increase in the friction coefficient, torsion angle, and the number of broken wires but will extend with the increase in temperature. The stress changes in the steel strand are as follows: when the number of broken wires increases, the maximum stress increases significantly and the average stress decreases slightly; when the temperature rises, the maximum stress and average stress in both cases of wire breakage show a significant linear decrease. These research results provide important references for the safety assessment and life prediction of cables in bridge engineering.

## 1. Introduction

Bridge stay cables are critical load-bearing components in cable-stayed bridges. Under repeated external conditions, wires within the cables may suffer damage or even fracture, compromising structural safety [1,2]. Therefore, investigating the wire breakage characteristics of stay cables is significant.

When the cable is corroded, the surface and internal microstructure of the cable will change significantly, the internal iron atoms preferentially dissolve and leach out, leading to voids or microcracks at the grain boundaries and defects [3], increasing the brittleness of the steel wire [4], reducing the fatigue life [5] and strength [6] of the steel wire, and then affecting the load-bearing capacity and structural safety of the cable. In addition, researchers have found that the influence of steel wire corrosion on the performance of the steel wire is greatly related to the stress size [7], stress ratio [8], corrosion pit parameters [9], anti-corrosion coating [10], etc. Under the coupling action of corrosion and fatigue, the corrosion medium and fatigue load promote each other, which will lead to a significant reduction in the performance of the steel wire [11]. To further explore factors influencing wire fatigue behavior, extensive studies have revealed that initial defects [12], contact forces [13], tensile stress [14], and traffic loads [15] all affect wire fatigue life.

In addition to external corrosion, contact wear between steel wires will cause spiral wear scars on the surface of steel wires [16], which will further reduce the mechanical properties of steel wires under the coupling action of friction and corrosion [17]. In addition, studies have found that the contact shape [18] and temperature [19] between steel wires will affect the degree of wear, thereby affecting the mechanical properties of steel wires. In order to better ensure structural safety and accurately identify and locate cable damage, many scholars have carried out related research. Based on noise emission [20], deep learning [21], and leakage magnetic field theory [22], a steel-wire damage-monitoring method is established to realize abnormal diagnosis of the cable state.

In order to further understand the mechanical properties of steel strands after broken wires, many scholars have conducted in-depth studies using different methods. Yu et al. [23]—based on theoretical methods and finite element models—calculated the stress recovery length of steel wires after broken wires and studied the stress redistribution mechanism of sections after broken wires. Based on the structural mechanics model, Xu et al. [24] developed a static model and derived the in-plane free-vibration control equations for damaged wire ropes. They also investigated the influence of wire breakage on the static and dynamic properties of wire ropes. Zhang et al. [25] carried out a bending–fatigue test on pre-broken wires. They discovered that wire breakage elevated the stress in internal wire strands and the contact stress among wires. It also hastened the growth of local wire–break density and shortened the bending–fatigue life of the wire rope. Wu et al. [26,27] studied the mechanical behavior of symmetrical and asymmetrical broken wires, established theoretical analysis models for the two broken wire situations, and studied the change in internal stress of the broken wire.

At present, the research focus of many scholars is mainly on the analysis of the cause of steel strand fracture failure and the application of the broken wire detection system, while research on the change law of the internal steel wire stress distribution after steel strand fracture is relatively small. At the same time, the in-depth study of the influence of various factors on steel wire fracture is also insufficient. In addition, when discussing the performance of steel strands, scholars usually rely on theoretical models and experimental analysis but relatively few simulation studies are carried out on the construction of finite element models—in particular, the study of models considering the interaction between steel wires is lacking. Studying the mechanical properties of steel strands after wire fracture is essential for evaluating structural safety and optimizing design, which helps to prevent structural failure and improve engineering reliability and durability. Steel strands are a common basic form of semi-parallel and parallel steel cables, and in-depth study of their basic characteristics can lay a solid foundation for the study of semi-parallel and parallel steel cables. Therefore, it is of great significance to study the broken wire characteristics of seven-wire steel strands.

This paper develops a finite element model for symmetrical and asymmetrical wire breaks in seven-wire steel strands. It investigates the internal force redistribution mechanisms following symmetrical and asymmetrical wire breaks in strands and examines the influences of temperature, friction coefficient, and torsion angle on break behaviors.

## 2. Finite Element Model

### 2.1. Construction of Finite-Element Beam-Element Model

The seven-wire steel strand adopts cold-drawn high-carbon steel as the base material, with a hot-dip galvanized surface treatment on individual wires. This wire type is an ultra-high-strength steel with a very high yield strength and is specially used for bridge cables, lifting systems, and other heavy load scenarios, which need to meet the requirements of high strength, corrosion resistance, and fatigue resistance. The dimensions of the seven-wire steel strand are based on the size specifications outlined in the Utting and Jones test [28]. Table 1 shows the geometric parameters and material parameters. In this study, a bilinear hardening criterion is used for the constitutive model of the steel wire.

Utilize ANSYS Classic software (version R18.2, ANSYS, Inc., Canonsburg, PA, USA) to create a parametric finite element model of a seven-wire steel strand beam element. The modeling process of the finite-element beam-element model for seven-wire steel strands is as follows: First, the geometric model of the central steel wire is created. Subsequently, a helical curve is generated around the central steel wire. After curve creation, 41 points are marked on the curve, and the SPLINE command is used to fit the helical curve through these key points, dividing it into multiple segmented helical curves. A circular cross-section for the lateral wire is then created on the working plane perpendicular to the curve. The Volume DRAG (VDRAG) command is employed to extrude the surface along the curve, forming a single lateral steel wire. Finally, the lateral wire is replicated using a loop command to create six lateral wires, which, together with the central wire, form the geometric model of the seven-wire steel strand.

To ensure the accuracy of analysis and computational efficiency, a finite-element beam-element model of seven-wire steel strands is established for subsequent analysis. The finite-element beam-element model of steel strands means that the steel wires in the steel strand cables are simulated by beam elements. In this paper, the Beam188 element is adopted, which is a three-dimensional linear finite-strain beam element and can be used to analyze slender beam structures.

Thereafter, the solid geometric model of the seven-wire steel strand is imported into ANSYS Workbench (version: R18.2, ANSYS, Inc., Canonsburg, PA, USA). Contact pairs are identified via the automatic contact pair detection function, and the beam element model is applied according to the element positions. The contact element between each beam element is Conta176, and the target element is Targe170. This element can simulate the line-to-line contact of the Beam188 element well. In addition, Coulomb friction is used to simulate the friction behavior between beam elements, supporting issues such as large sliding and large slip. Large deformation effects were enabled globally to capture geometric nonlinearity.

The contact element settings within the model are as follows: Set the key option 3 (KEYOPT (3)) to 0. The contact offset (CNOF) parameter is designed to eliminate the gap between the contact surface and the target surface. Here, the automatic CNOF is used to close the gap. The time step control is configured such that when the contact state changes, the contact predictor determines the minimum time step or load increment; contact stiffness is updated at each substep; and the contact surface behavior is set to “non-separation with allowable sliding”. The intrusion tolerance coefficient FTOLN is set to 0.05, and the maximum contact friction stress TAUMAX is specified as σs/3, where σs refers to the material yield strength. Use the command stream to define the material properties of the steel wire. The material property parameters of the steel are shown in Table 1.

**Figure 1:**
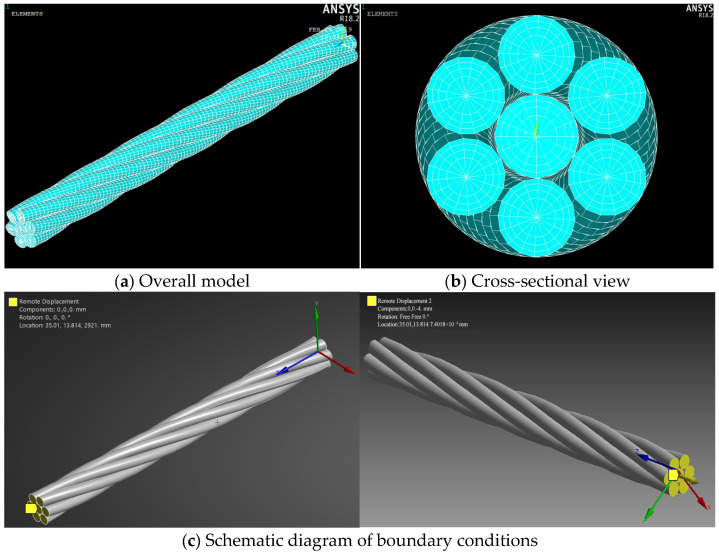
Finite-element beam-element model.

For meshing, default settings in the “Mesh” module are adopted, using the sweep meshing method with 40 segments along the length direction. “Contact sizing” is inserted to refine the mesh in contact regions, with the refined element size set to 3 × 10^−4^ m. The ANSYS Workbench default Solid186 element is used for meshing. When setting the boundary conditions, first apply a fixed constraint to the nodes at one end. Then, to simulate the anti-rotation fixture in the actual anchor cable, constrain the rotational degree of freedom in the *Z*-direction at the nodes at the other end and apply the corresponding displacement load. Automatic time stepping is enabled, the Newton–Raphson method is set to “Full”, and linear search is used. The resulting finite-element beam-element model is shown in Figure 1a. The cross-sectional view of the model is shown in Figure 1b, displaying the baseline mesh for intact strands, and the wedge-shaped element near the center of the steel wire is the Conta176 contact unit, which is paired with Targe170 target unit to simulate the contact pressure and friction behavior between steel wires. The schematic diagram of boundary conditions is shown in Figure 1c.

### 2.2. Validation of the Finite Element Model

**Figure 2:**
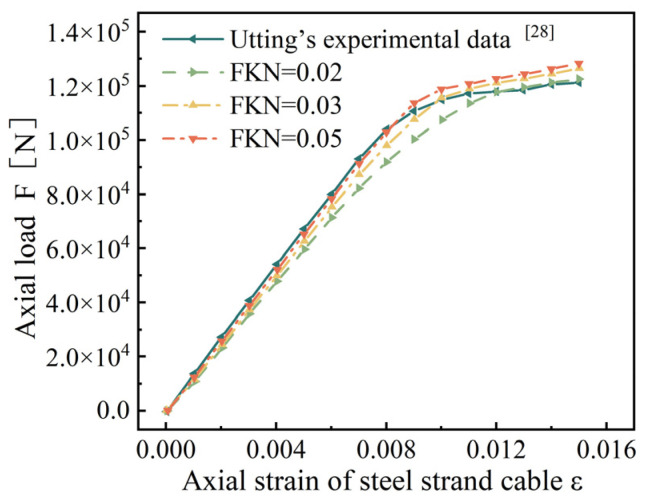
Axial strain–load curve. (Different FKN values and Utting’s experimental data [28]).

Because the normal contact stiffness coefficient (FKN) has a great influence on the results of the bearing reaction force, FKN is taken as 0.02, 0.03, and 0.05, respectively, for discussion [29], and the relationship curve between the axial strain and load is drawn, as shown in Figure 2.

Figure 2 shows that as FKN increases, the finite element results in the elastic stage approach closer and closer to Utting’s experimental data. However, when the axial strain exceeds 0.008 and the steel strand enters the plastic stage, the finite element results deviate from Utting’s experimental data. After comprehensive consideration, we choose FKN = 0.05 for the subsequent research.

**Figure 3:**
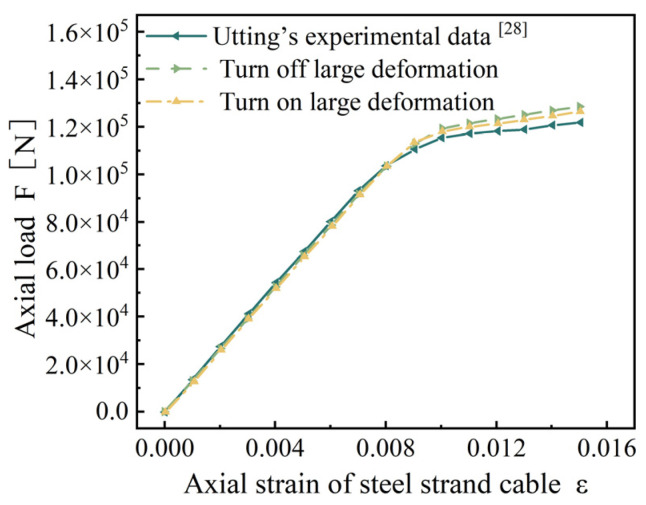
Axial strain–load curve. (Large deformation and Utting’s experimental data [28]).

Considering the influence of large deformation on the results, the simulation results are compared with Utting’s experimental results, see Figure 3. After calculation, the maximum error between the results after opening the large deformation setting and Utting’s test results is 4.30%. Therefore, we choose to open the large deformation setting for subsequent research.

## 3. Finite Element Study on Wire Breaking Characteristics

### 3.1. Symmetrical Broken Wires

**Figure 4:**
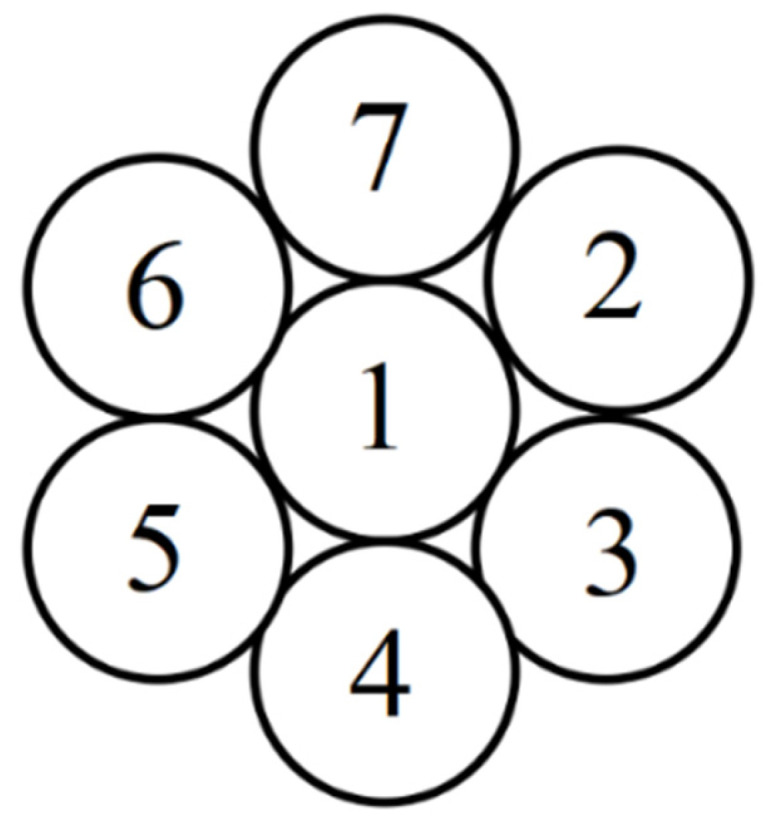
Steel wire numbering diagram.

**Figure 5:**
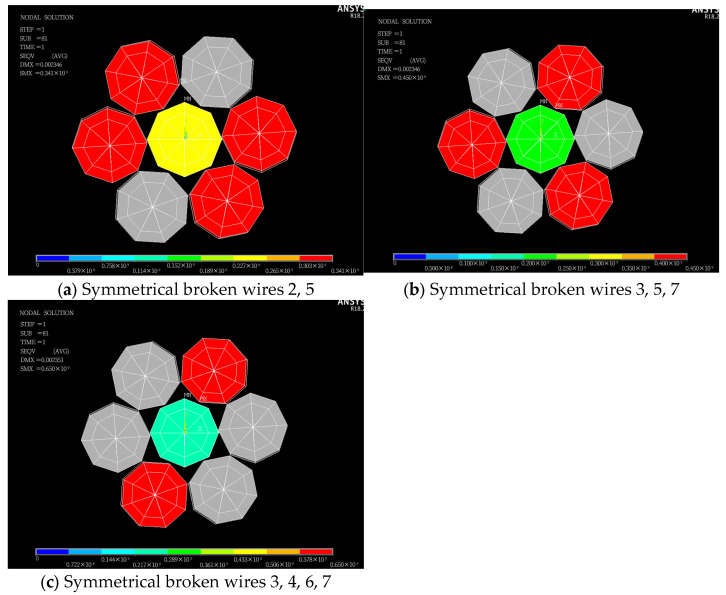
Stress contour plot of broken wires after symmetrical broken wire (unit: Pa).

The steel wire numbering is shown in Figure 4. In this study, “broken wire failure” refers to the complete breakage of the steel wire at this cross-section, resulting in the loss of load-bearing capacity. First of all, the situation of symmetrical broken wire is studied. A symmetrical broken wire refers to the situation where the broken wire position presents geometric symmetry distribution on the cross-section of the steel strand. There are three kinds of symmetrical broken wires of seven-wire steel strands: steel wires 2 and 5 fracture; steel wires 3, 5, and 7 fracture; and steel wires 3, 4, 6, and 7 fracture. In the ANSYS software, the element birth and death command can simulate material failure. To simulate the broken wire failure of the steel strand, at the mid-span position of the steel strand, the element-birth-and-death technique is used to simulate the sudden fracture failure of the steel wire. In addition, the stiffness loss after the steel wire breaks is handled through the element inactivation function. A locally refined mesh is presented near the fracture area to capture the stress gradient, as shown in Figure 5.

It can be seen from Figure 5 that when the wire is symmetrically broken, the stress of each wire at the broken wire is symmetrically distributed. The stress of each intact side wire is roughly the same, and the stress of the side wire is higher than that of the central wire. The gaps between the steel wires in the figure are caused by the visualization limitations of the beam contact element.

**Figure 6:**
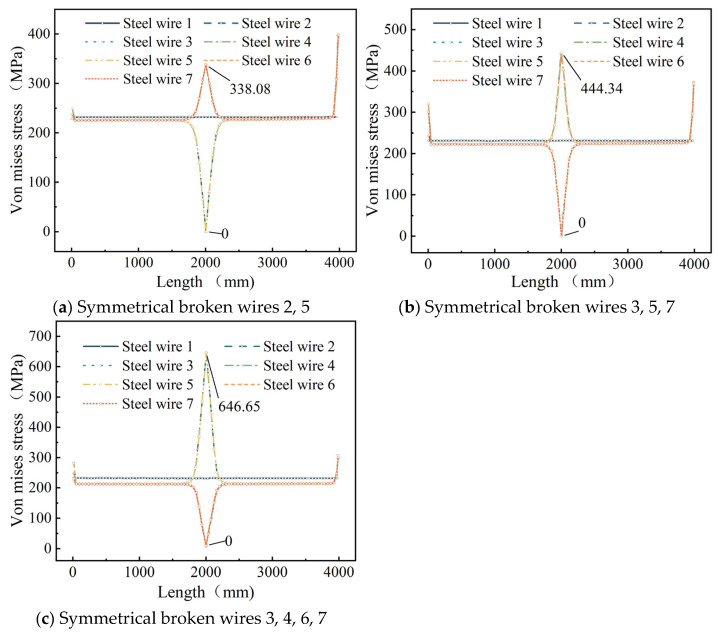
Stress redistribution of seven-wire steel strands after symmetrical broken wire.

In order to further study the stress redistribution in the cable after the steel strand is broken, the stress distribution diagram of the steel wire along the length of the steel wire is drawn, as shown in Figure 6.

As illustrated in Figure 6, under symmetrical wire breakage in the steel strand, the stress variation in each broken wire is identical. Similarly, the stress variation in each intact peripheral wire also follows a uniform pattern. At the breakage cross-section, the stress in the broken wires rapidly decreases to zero, while the stress in the remaining intact peripheral wires increases abruptly. The stress in the central wire exhibits negligible variation along its entire length, with stresses symmetrically distributed on both sides of the breakage cross-section. An abrupt cross-sectional change at the wire’s anchored end creates geometric discontinuity, causing distinct stress peaks at L = 0 and 4000 mm per elastic mechanics theory.

When wires 2 and 5 are severed, the stress in the remaining intact peripheral wires increases exponentially from 226.11 MPa to 338.07 MPa, representing a 49.6% increase. For the cases of broken wires 3, 5, and 7, the stress in the intact peripheral wires undergoes a rapid exponential rise from 222.12 MPa to a peak of 444.34 MPa, corresponding to a 100% increase. When wires 3, 4, 6, and 7 rupture, the stress in the intact peripheral wires surges exponentially from 213.44 MPa to 646.65 MPa, marking a 203.3% elevation.

**Figure 7:**
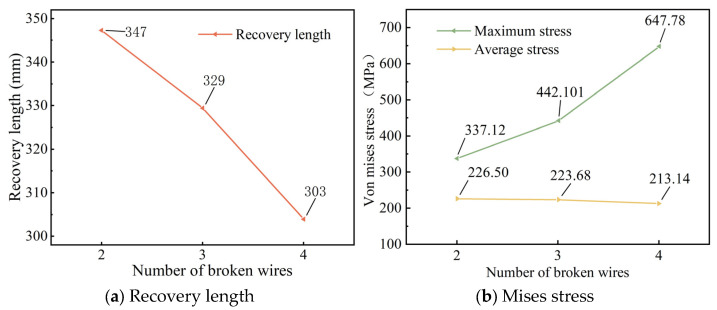
Effect of the number of broken wires.

Figure 7 illustrates the effect of the number of broken wires on the stress recovery length of the seven-wire steel strand and the stress in its steel wires. The maximum stress is defined as the peak stress on the unbroken steel wire, while the average stress refers to the stress in steel wires outside the recovery length.

Figure 7a shows that increasing the number of broken wires significantly shortens the stress recovery length. As the number of broken wires increases from 2 to 4, the recovery length decreases from 347 mm to 303 mm. As the quantity of broken wires rises, the shear force among intact steel wires grows stronger, leading to a quicker transfer of stress and, consequently, a reduced recovery length [23]. Figure 7b indicates that the number of broken wires significantly affects the maximum stress but barely influences the average stress. As the number of broken wires increases from 2 to 4, the maximum stress of the steel strand rises from 337.12 MPa to 647.78 MPa, representing an increase of 92.2%.

### 3.2. Asymmetrical Broken Wires

**Figure 8:**
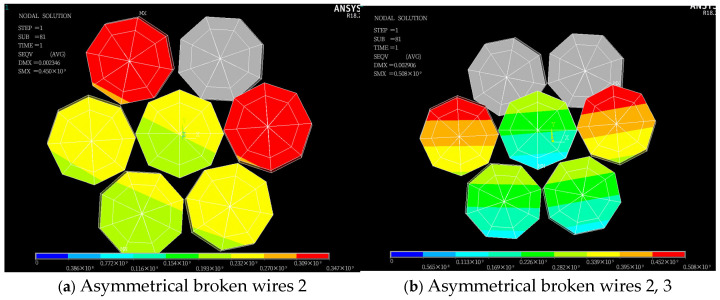

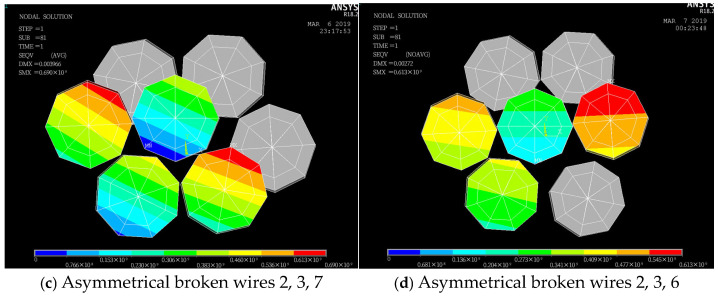
Stress contour plot of broken wires after asymmetrical broken wire (unit: Pa).

An asymmetrical broken wire refers to the situation where the position of the broken wire does not show geometric symmetry distribution on the cross-section. This kind of broken wire usually leads to an unbalanced stress of steel strands and produces additional bending moments. Typical asymmetric modes include single-peripheral-filament breakage, multiple-adjacent-peripheral-filament breakage, and discontinuous-multiple-peripheral-filament breakage. Four types of asymmetrical wire failure cases in the seven-wire steel strand are defined: failure of steel wire 2; failures of steel wires 2 and 3; failures of steel wires 2, 3, and 7; and failures of steel wires 2, 3, and 6. Stress contour maps for the strands following asymmetrical wire failures are presented in Figure 8, which shows locally refined meshes near fracture zones to capture stress gradients.

Figure 8 shows that following asymmetric wire failure, the stress of the side wires adjacent to the broken wire in the steel strand cable is significantly higher than that of other steel wires. The stress in each steel wire is no longer symmetrically distributed along their central axes. Furthermore, the asymmetric stress distribution disrupts the original force balance, causing the entire steel strand to undergo asymmetric torsional deformation. The torsional deformation of each steel wire results in an overlap among the steel wires on the cross-section.

**Figure 9:**
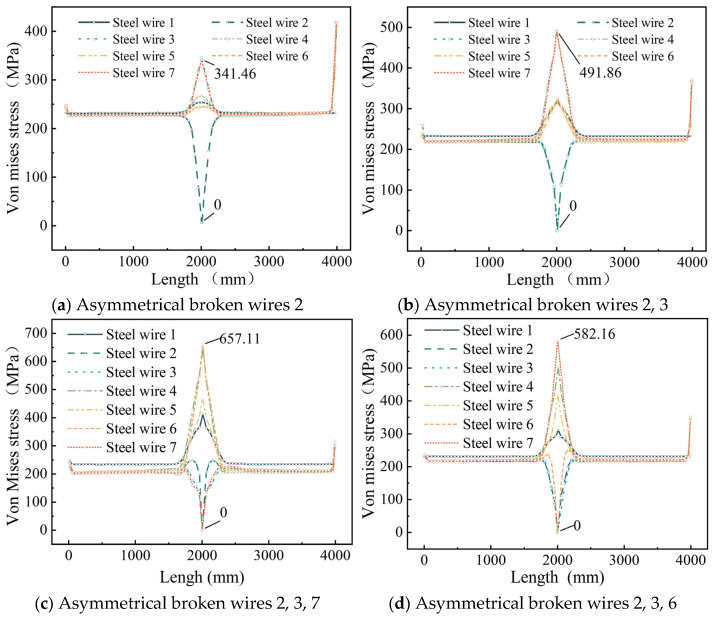
Stress redistribution of seven-wire steel strands.

To further investigate stress redistribution in the steel strand following asymmetric wire failure, we plotted the stress distribution diagram of steel wires along their length (Figure 9).

Figure 9 shows that, unlike symmetrical wire failures, the longitudinal stress changes in each intact adjacent wire differ following asymmetrical wire failure in the strand. After wire failure, the stress at the failure section of the broken wire rapidly drops to zero, while the stress in the remaining adjacent intact wires increases sharply, with the maximum stress occurring in the intact wires adjacent to the failure. In addition, the stress of the central wire also increases suddenly at the broken wire section.

When steel wire 2 breaks, the stress in adjacent steel wires 3 and 7 increases from 228.13 MPa to a peak of 341.46 MPa, representing a 49.6% increase; when wires 2 and 3 break, the stress in adjacent wires 4 and 7 rises from 224.21 MPa to a peak of 491.86 MPa, resulting in a 119.2% increase; when wires 2, 3, and 7 break, the stress in adjacent wires 4 and 6 increases from 222.41 MPa to a peak of 657.11 MPa, reflecting a 195.9% increase; when wires 2, 3, and 6 break, the stress in adjacent wire 7 increases from 221.42 MPa to a peak of 582.16 MPa, corresponding to a 163.3% increase.

## 4. Parameterized Sensitivity Analysis

With the aim of exploring the influence mechanisms of steel wire internal parameters, temperature, friction coefficient, and strand twist angle on the fracture of symmetric and asymmetric steel wires within a seven-wire strand, the present study selects two representative scenarios: symmetric breakage of wires 2 and 5, and breakage of wires 2 and 3.

### 4.1. Effect of Temperature

**Figure 10:**
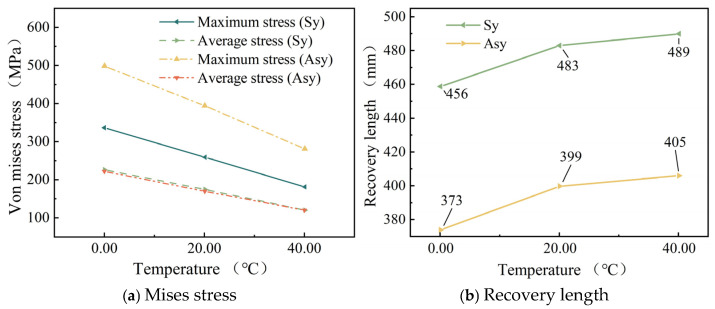
Effect of temperature.

The relationship between temperature, the maximum stress, and the average stress in the strands is shown in Figure 10a, and the relationship between temperature and the recovery length of the broken wire stress is shown in Figure 10b. In the subsequent figures, Sy represents a symmetric broken wire, and Asy represents an asymmetric broken wire.

Figure 10a shows that as the temperature rises from 0 °C to 40 °C, both the maximum and average stresses of strand wires exhibit a linear decrease. For both symmetric and asymmetric wire breaks, the average stress values are nearly identical. In terms of maximum stress, asymmetric wire breaks exhibit significantly higher values than symmetric ones. Under symmetrical wire breakage, as the temperature increases from 0 °C to 40 °C, strand maximum stress decreases from 336.11 MPa to 180.35 MPa, and average stress drops from 223.43 MPa to 120.37 MPa. Under asymmetric wire breakage, strand maximum stress decreases from 498.14 MPa to 281.41 MPa, with average stress declining from 221.13 MPa to 121.24 MPa.

Figure 10b shows that a temperature rise causes the stress recovery length of the strand wires to increase, with roughly similar growth trends in recovery length under both breakage scenarios. When the temperature increases from 0 °C to 20 °C, the effect of temperature rise on recovery length is more pronounced. As the temperature increases further, the rate at which recovery length increases gradually diminishes.

### 4.2. Effect of Friction Coefficient

**Figure 11:**
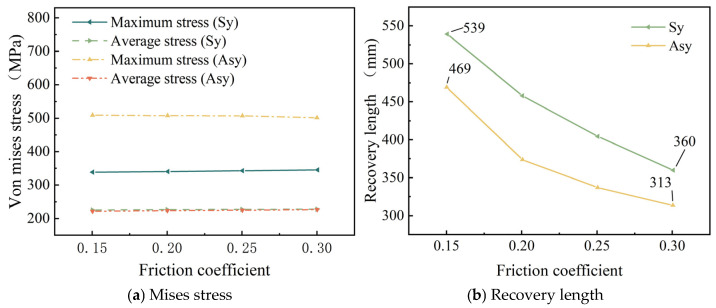
Effect of friction coefficient.

The friction type between steel wires is dynamic friction. The parameter range is demonstrated by the data from the cable industry [13,17]. In order to study the influence of friction coefficient on symmetrical and asymmetrical wire breakage of seven-wire steel strands, the relationship between friction coefficient and maximum stress and average stress in steel strands is summarized as Figure 11a, and the relationship between friction coefficient and stress recovery length of broken wire is summarized as Figure 11b.

Figure 11a shows that the increase in the friction coefficient has a relatively small effect on the internal stress variation in the seven-wire steel strand when it breaks. In contrast, Figure 9b reveals a pronounced effect of the friction coefficient on post-breakage stress-recovery length. Notably, when the friction coefficient is low, the recovery length decreases significantly as the friction coefficient increases. For symmetric wire breakage, increasing the friction coefficient from 0.15 to 0.30 reduces the recovery length from 539 mm to 360 mm—a 33.4% decrease. For asymmetric wire breakage, the recovery length decreases from 469 mm to 313 mm—a 33.2% decrease.

**Figure 12:**
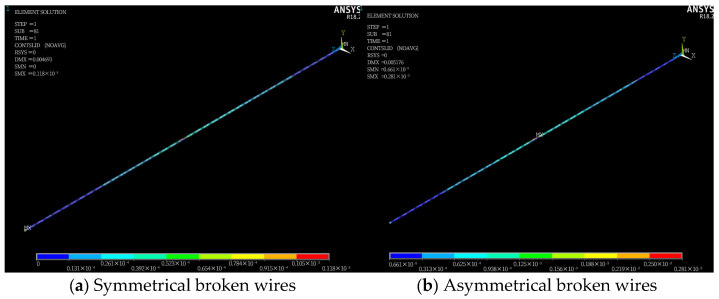
Wire slip distribution (unit: mm).

The distribution of wire slippage along the length after the steel strand breaks is shown in Figure 12.

As can be seen from Figure 12, the maximum slip distance of symmetrical broken wires occurs at the loading end but a relatively large slip also exists at the wire breakage position; the maximum slip distance of asymmetrical broken wires occurs at the broken wire position.

**Figure 13:**
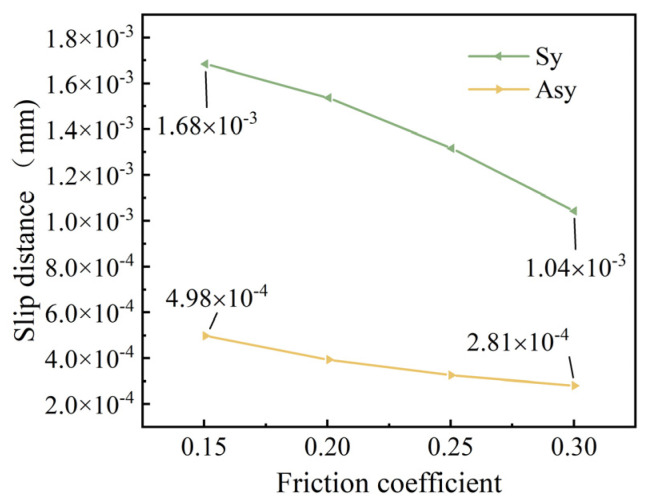
Relationship between friction coefficient and slip distance.

The relationship between the maximum slip distance of the steel wire and the friction coefficient of the steel wire is shown in Figure 13.

As can be seen from Figure 13, under the same friction coefficient, the slip distance for symmetric broken wire is significantly higher than that for asymmetric broken wire. When the friction coefficient increases from 0.15 to 0.30, the maximum slip distance during symmetric broken wire decreases by 38.1%, while the maximum slip distance during asymmetric broken wire decreases by 43.6%.

### 4.3. Effect of Torsion Angle

**Figure 14:**
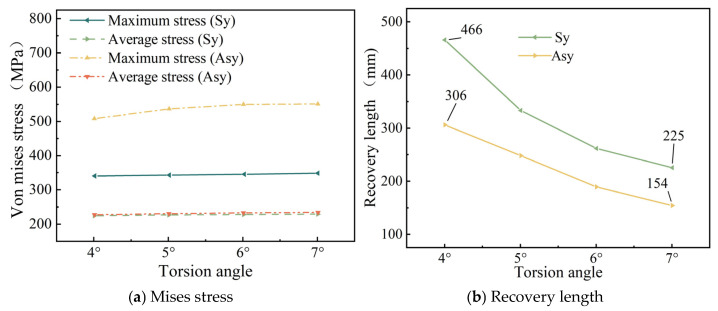
Effect of torsion angle.

Figure 14a illustrates the relationships between torsion angle, strand maximum stress, and average stress, while Figure 14b depicts the relationship between torsion angle and post-breakage stress-recovery length.

Figure 14a shows that the torsion angle has a limited effect on strand internal stress. Figure 14b indicates that as the torsion angle increases, the stress recovery length decreases significantly under both symmetric and asymmetric wire breakage, with symmetric breakage exhibiting a longer recovery length than asymmetric breakage. As the torsion angle increases from 4° to 7°, the recovery length decreases by 51.6% for symmetric wire breakage and 49.7% for asymmetric wire breakage.

## 5. Discussion

Based on the finite-element beam-element model of seven-wire steel strands, this study establishes a fine finite element model for symmetrical and asymmetrical wire breaks in seven-wire steel strands to investigate the wire-breaking performance of strands. The model’s validity was confirmed via the following: Utting’s replication test (Figure 2 and Figure 3, error < 4.3%); theoretical alignment: recovery length vs. Wu’s model [26] (deviation < 7%); stress concentration vs. Yu’s FEM [23] (R^2^ = 0.94).

As depicted in Figure 6 and Figure 9, when a seven-wire steel strand experiences wire breakage, stress redistribution takes place within the broken wire segment. The stress of the broken wire rapidly drops to zero, and the stress of the remaining intact wires on the side increases sharply due to the failure of the broken wire. The maximum increase can reach up to 203.3%, thereby increasing the risk of complete fracture of the side wires. Wu et al. [26,27] constructed a theoretical model of steel strand broken wire, compared it with other experimental results to demonstrate the theoretical model, and obtained similar results. Yu et al. [23] combined the theoretical model and finite element analysis to further verify this conclusion.

As for the stress recovery length of broken wires, Figure 7a shows that as the number of broken wires increases, the stress recovery length of broken wires decreases significantly. Next, Figure 10b indicates that as the temperature rises, the stress recovery length of the wires exhibits an upward trend. Moreover, as the temperature continues to increase, this upward trend gradually slows down. Additionally, Figure 11b reveals that as the friction coefficient increases, the stress recovery length of the wires decreases significantly. This implies that the friction effect of the adjacent wires promotes the recovery of the broken wire stress. Figure 14b demonstrates that as the torsion angle of the wires increases, the stress recovery length of broken wires decreases.

Based on the established theoretical mechanical model, Wu et al. [26,27] also explored the impacts of the number of broken wires and the contact friction among wires on the stress recovery length. The conclusions they reached are consistent with those of this paper. Yu et al. [23]—based on theoretical calculations and finite element simulation analysis—also pointed out that the recovery length is related to the twist angle of the wire and the friction coefficient between the wires, and the influence trend is similar to that of this paper. In addition, by comparing the above data graphs, it can be seen that under the same conditions, the stress recovery length under a symmetrical broken wire is significantly higher than that under an asymmetrical broken wire.

Additionally, this paper conducts a parameter analysis of the stress changes in the wires of a seven-wire steel strand after wire breakage. According to Figure 7b, as the number of broken wires increases, the maximum stress within the seven-wire steel strand increases significantly, while the average stress decreases to some extent. This indicates that an increase in the number of broken wires decreases the load-bearing capacity of the steel strand. The load on the intact wires rises continuously. This situation is increasingly detrimental to the intact wires and may even result in the failure of the unbroken wires. Figure 10a shows that as the temperature rises, both the maximum and average stresses within the steel strand decrease significantly in a linear manner for both scenarios of symmetric wire breakage and asymmetric wire breakage. Moreover, by comparing the aforementioned figures, it can be noted that under the same conditions, the average stress values of the wires in both symmetric and asymmetric wire breakage cases are nearly identical. However, for the maximum stress of the wires, the values in the asymmetric case are significantly higher than those in the symmetric wire breakage case. Wu et al. [27] proposed that an asymmetrically fractured strand would deflect laterally to the fractured strand while a symmetrically fractured strand would not. The lateral deflection caused by this lateral deflection will increase the stress of the unbroken wire. This theory explains the above situation very well.

It should be pointed out that the analysis of temperature effect in this study has some limitations. It is assumed that the temperature field is evenly distributed and the fluid-structure coupling mechanism is not included. These simplified treatments may affect the accuracy of the sensitivity analysis of temperature parameters, and a more perfect thermal–mechanical coupling model will be established in the follow-up study for further exploration.

## 6. Conclusions

Based on the finite-element beam-element model, this paper constructs models for symmetric and asymmetric wire breaks in seven-wire steel strands and derives the stress redistribution mechanism following the breakage of these strands. Subsequently, the influences of temperature, friction coefficient, and torsion angle on symmetric and asymmetric wire breaks in seven-wire steel strands are examined. The key conclusions are summarized as follows:

1. Once the steel wire in the steel strand breaks, the stress of the broken wire at the fracture site will rapidly drop to zero, while the stress of the intact adjacent wires at the fracture site will quickly reach its peak value. When symmetrical wire breaks occur in wires Nos. 3, 4, 6 and 7, the maximum stress increase reaches 203.3%. In the case of symmetric wire breakage, the law of stress change for each intact adjacent wire along the axial direction is identical. Conversely, in the case of asymmetric wire breakage, the stress changes in each adjacent wire are not completely identical. Among these, the stress of the adjacent wire to the broken wire is significantly higher than that of the other wires.
2. The increase in the number of broken wires, the increase in the friction coefficient, and the increase in the torsion angle of the steel wires will all significantly reduce the stress recovery length of the broken wires. When the friction coefficient increases from 0.15 to 0.30, the corresponding stress recovery length is shortened by 33.2%. When the torsion angle increases from 4° to 7°, the stress recovery length is reduced by 51.6%. When the temperature rises, the recovery length will increase, and as the temperature increases, the growth trend gradually slows down. In addition, under the same conditions, the stress recovery length in the case of symmetrically broken wires is significantly higher than that in the case of asymmetrically broken wires.
3. With the increase in the number of broken wires, the maximum stress within the steel strand increases significantly, while the average stress decreases. Under the two working conditions of broken wires, as the temperature rises, both the maximum stress and the average stress within the steel strand decrease significantly in a linear manner. When the temperature increases from 0 °C to 40 °C, the stress decreases by 77.2%. The influence of the torsion angle on the stress within the steel strand is relatively limited. Only when the symmetrical wire breakage occurs will the increase in the torsion angle lead to a significant increase in the maximum stress within the steel strand. In addition, under the same conditions, the average stress values of the steel strand in the two cases of broken wires are very close, while for the maximum stress, the value under the asymmetric condition is significantly higher than that under the symmetrical wire breakage condition.

Based on the existing conclusions, the following aspects can be further studied in the future: optimizing the torsion angle and contact geometry of steel wires to minimize wear and stress concentration caused by slip; researching functional coatings and controlling the temperature to dynamically adjust the stress of steel wires; using the asymmetric wire breakage model as the worst-case scenario to verify the strength redundancy of steel wires; and carrying out multi-physical field modeling to establish a complete thermal–structural coupling model for further exploration.

## Figures and Tables

**Table 1:** Main parameters of seven-wire steel strand.

Steel Wire Diameter/mm	Lay Length/mm	Model Length/mm	Elastic Modulus/MPa	Yield Strength/MPa	Tangent Modulus/MPa	Poisson’s Ratio	Coefficient of Friction
3.5	629	4000	1.982 × 10^5^	1.54 × 10^3^	24.6 × 10^3^	0.27	0.2

## Data Availability

The original contributions presented in this study are included in the article. Further inquiries can be directed to the corresponding author.
